# Bioaugmentation potential of free and formulated 2,6-dichlorobenzamide (BAM) degrading *Aminobacter* sp. MSH1 in soil, sand and water

**DOI:** 10.1186/s13568-016-0204-1

**Published:** 2016-04-30

**Authors:** Nadja Schultz-Jensen, Jens Aamand, Sebastian R. Sørensen

**Affiliations:** Department of Geochemistry, Geological Survey of Denmark and Greenland (GEUS), Øster Voldgade 10, 1350 Copenhagen K, Denmark

**Keywords:** Dichlobenil, Bacteria formulation, Bioremediation, Bioaugmentation

## Abstract

Pesticides are used extensively worldwide, which has led to the unwanted contamination of soil and water resources. Former use of the herbicide 2,6-dichlorobenzonitrile (dichlobenil) has caused pollution of ground and surface water resources by the stable degradation product 2,6-dichlorobenzamide (BAM) in several parts of Europe, which has resulted in the costly closure of several drinking water wells. One strategy for preventing this in future is bioaugmentation using bacterial degraders. BAM-degrading *Aminobacter* sp. MSH1 was therefore formulated into dried beads and tests undertaken to establish their potential for use in the remediation of polluted soil, sand and water. The formulation procedure included freeze drying, combined with trehalose addition for cell wall protection, thus ensuring a high amount of viable cells following prolonged storage at room temperature. The beads were round-shaped pellets with a diameter of about 1.25 mm, a dry matter content of approximately 95 % and an average viable cell content of 4.4 × 10^9^ cells/g bead. Formulated MSH1 cells led to a similar, and frequently even faster, BAM mineralisation (20–65 % ^14^CO_2_ produced from ^14^C-labelled BAM) in batch tests conducted with sand, water and different soil moisture contents compared to adding free cells. Furthermore, the beads were easy to handle and had a shelf life of several months.

## Introduction

Organic herbicides are used worldwide in agricultural crop production for protection against weed, insect and fungal attack. The production of pesticides, as well as their use, has led to the unwanted contamination of soil and water resources, and in many cases remediation measures are required to protect the environment and secure drinking water resources. Former use of the herbicide 2,6-dichlorobenzonitrile (dichlobenil) has led to the pollution of surface and groundwater resources by the degradation product 2,6-dichlorobenzamide (BAM). As a direct consequence, several drinking water wells have been closed due to the EU threshold values for pesticides in drinking water being exceeded. In Denmark, for example, BAM has been detected in 20 % of groundwater monitoring wells and has been above the 0.1 µg L^−1^ threshold limit in approximately 10 % of the wells (Thorling et al. 2011).

Microorganisms play an essential role in remediation of most organic contaminants in soil and water resources due to their metabolic versatility and their capacity to adapt to changing environmental conditions (Meckenstock et al. 2015). However, their efficiency is determined by the pollutants chemical structure and concentration—along with the bioavailability and the physiochemical characteristics of the affected environment (Karlson et al. 1995; Fantroussi and Agathos 2005). The capacity of indigenous microorganisms to degrade organic pollutants within an environment matrix can however be enhanced, either through stimulation by adding substrates and nutrients for example, or by the addition of contaminant-degrading microorganisms (Karlson et al. 1995; Sheehan 1997). The last strategy, called bioaugmentation, is a relevant remediation approach when indigenous populations lack the capacity to degrade the contaminant. It has also been suggested as an economically feasible and environmentally friendly method for contaminant remediation (Fantroussi and Agathos 2005; Thompson et al. 2005; Benner et al. 2013).

Bioaugmentation is not a straightforward approach, however, and the technique is in its infancy and needs to be developed into a mature and validated method. In numerous cases, the introduction of specific bacteria into a foreign natural environment has had a minor effect on degradation efficiency. This have been explained by overlapping phenomena such as competition for nutrients and resources, predation by natural microorganisms and the lack of ability to sustain activity and viability for longer periods (Steenson et al. 1987; Acea et al. 1988; Compeau et al. 1988; Knaebel et al. 1997; Gentry et al. 2004; Owsianiak et al. 2010). The simplest bioaugmentation approach is the addition of the inocula in a liquid phase. In laboratory evaluations, however, more advanced strategies using immobilised degraders have proven superior (Moslemy et al. 2006; Siripattanakul and Khan 2010) and this could be a way of improving bioaugmentation efficiency.

Specific organisms for introduction into the environment have been combined with different types of carrier materials, such as agar, peat, alginate, alginate-clay and fluid gels (Stromo and Crawford 1994; Petrich et al. 1998). Some carriers, for example, allow a high concentration of cells to be incorporated into the contaminated environment, and this might act as a hot spot with a slow release of viable cells into the surrounding environment (Mertens et al. 2006). Another similar strategy is to formulate viable cells into solid dried forms, which could be stored for longer periods and repeatedly introduced into contaminated environments of interest. Such formulated cell products could, for example, also contain nutrients and be designed to protect cells against high concentrations of toxic compounds (Stromo and Crawford 1994; Petrich et al. 1998; Saez et al. 2012).

Previous studies on the BAM-mineralising bacterium *Aminobacter* sp. strain MSH1 have been conducted with free cells in laboratory batch or column tests with soil and water (Sørensen et al. 2007; Simonsen et al. 2012; Albers et al. 2014). The current study included the development of a novel formulation technique for the immobilisation of MSH1, aimed at producing an easy-to-handle bead product with a high density of viable cells. The formulation procedure included freeze drying combined with trehalose addition for cell wall protection. This study was based on the recent development of a fermentation protocol for large-scale production of MSH1 cells (Schultz-Jensen et al. 2014), providing sufficient amounts of viable cells for technical application. Besides strain MSH1, the phenoxy acid herbicide-degrading *Sphingomonas* sp. strain PM2 was included in the bead production, but excluded in the later stages of the study based on lack of sustained viability.

## Materials and methods

### Microorganisms

The *Aminobacter* sp. strain MSH1 was previously isolated in the laboratory from contaminated soil using BAM as the sole carbon and nitrogen source (Sørensen et al. 2007) and deposited in the Pasteur collection (CIP 110285). The strain *Sphingomonas* sp. PM2, which is capable of degrading several phenoxy acid herbicides, was isolated in the laboratory and characterised by Johannesen (2002); it is a promising candidate for remediation of a broad range of herbicide concentrations (Qiu et al. 2014; Krüger et al. 2015). Both strains were stored in 40 % sterile glycerol at −80 °C and thawed and pre-cultivated before the experiments.

### Media and cultivation

The *Aminobacter* sp. strain MSH1 was cultivated in MSNC_opt_ medium, as previously described in Schultz-Jensen et al. (2014). *Sphingomonas* sp. PM2 was cultivated in the Oppermann medium (OPM) (Steinbüchel and Oppermann-Sanio 2003) supplemented with 0.2 % glucose and adjusted to pH 6.9.

Precultures and experimental cultures were made as previously described (Schultz-Jensen et al. 2014). Viable cells numbers were estimated by plating on R2A agar plates (Difco R2A agar, Becton–Dickinson and Company, Sparks, MD 21152 USA). The agar was stirred for 5 min and autoclaved for 20 min at 121 °C.

### Bacterial growth measurements

#### Drop plating

The method used for drop plating was that described by Herigstad et al. (2001). Serial dilutions were prepared in a mineral medium (MSNC_opt_ with no added carbon source) and five 10 µL drops were placed on the surface of the R2A plates. All plating work was performed in a laminar airflow bench and the plates were incubated at 20 °C for 4–5 days and the number of CFU (colony-forming units) counted.

#### Enumeration of bead cells

Single beads of the immobilised cells were weighed into Eppendorf tubes and 1 mL of MSN (MSNC_opt_ medium with no carbon source, iron and trace elements) was added. The sample was kept for 5 min under the laminar flow bench to dissolve the bead and release the immobilised bacteria. Thereafter, the sample was shaken by hand, vortexed thoroughly and viable cell numbers determined by drop plating.

#### Optical density (OD)

OD measurements were performed using a spectrophotometer (Jenway 6061 colorimeter, Herlev, Denmark). MSH1 was grown in MSNC_opt_ medium and PM2 in OPM medium, as described above. 2 mL samples were taken out of the cultivation flasks at regular intervals with a sterile pipette and OD_600 nm_ was measured. The change in pH was measured with a pH meter (PHM220 LAB pH METER, Radiometer, Copenhagen, Denmark). Samples were taken at regular time intervals and OD_600 nm_ was measured and drop plating performed. Based on the CFU/OD correlation, the same amounts of free and immobilised cells were added to batch flasks in the bioremediation experiment.

### Bead formulation

#### Bacteria-containing beads

The *Aminobacter* sp. strain MSH1 used for the immobilisation was grown under optimised conditions as described previously (Schultz-Jensen et al. 2014). Cells were grown in liquid medium to a dry matter content of ~1.7 g L^−1^. Freshly grown cells were centrifuged at 6000 rpm for 10 min before mixing with trehalose. The centrifuged cells had 15 % dry matter. 5 g cells were mixed with a trehalose solution of 80 % w/v, containing 3.5 g trehalose in 1 mL MSN medium. The mixture was vortexed thoroughly for 1–2 min and then pelleted to ensure that no settling of the cells had occurred during the incubation (Conrad et al. 2000). The beads were prepared by drop-wise addition of the cells and trehalose mixture using a syringe with a 23-gauge needle (Sterican 23 G × 1 ¼, Braun, Germany) into liquid nitrogen to freeze immediately into beads (Conrad et al. 2000). Bead size could be varied according to the needle size (for example a 25-gauge needle would produce a round bead size of ~2.5 mm, and a 23-gauge needle would produce a bead size of ~1.25 mm). The beads were than immediately transferred to the freeze drier (ScanVac CoolSafe Freeze Drying, LaboGene ApS, Denmark) and freeze-dried for 24 h. This resulted in uniform, round-shaped pellets approximately 1.25 mm in diameter, with an average weight of 0.08 g, a dry matter content of ~95 % and an average amount of 1.5 × 10^9^ CFU g^−1^ bead. Beads were stored in containers with CaCl_2_ at room temperature for several months. The *Sphingomonas* sp. strain PM2 used for the immobilisation was cultivated in the OPM medium and the beads were prepared as described for strain MSH1.

#### Cell-free beads

Beads without cells were prepared as a control. 6 g trehalose was mixed with 10 mL MSN medium, resulting in a 40 % w/v trehalose solution. The pellets were prepared by the drop-wise addition of trehalose mixture through a syringe into liquid nitrogen. The beads were immediately transferred to the freeze drier, as described above for beads containing cells. For the bioremediation experiment, the same weight of beads with and without cells was used. This ensured that the same amount of trehalose (a potential carbon source for bacteria) was added to each batch experiment.

### Bioaugmentation experiment

The testing of beads in a bioaugmentation scenario was only conducted with the MSH1 beads as the PM2 beads showed low survival following storage.

#### Material for the laboratory-scale test

Samples were collected from a sandy soil (Humic Podzol) in the western part of Jutland (9°0′31.00″E, 56°17′58.64″N), Denmark. This soil has previously been characterised (Albers et al. 2008). Different water contents were adjusted in the sand and soil samples. The highest water contents were obtained by adding 2 mL of MSN (MSNC_opt_ medium with no carbon source, iron and trace elements added) to 10 g of sand or soil. Soils with medium and low water contents were adjusted by adding 1 or 0.5 mL of MSN respectively to 10 g soil. The sand was purchased from Fisher Scientific (Leicestershire, UK) and had a particle size of 20–30 mesh.

#### BAM mineralisation

In total, 78 sterilised glass flasks with airtight stoppers were prepared containing free and immobilised cells in water (MSN medium), sand (high water content) or soil (high, medium and low water contents). Each batch flask was supplied with either 12 mL buffer or 10 g sand or soil respectively. Subsequently, the mineralisation experiment was initiated by the addition of a mixture of unlabelled and ^14^C-labelled BAM and MSH1 cells, as described below. Cell-free beads were included in the experiment as a control. All flasks were incubated for 36 days at 20 °C in the dark and sampled at regular time intervals.

Mineralisation experiments were used as previously described (Schultz-Jensen et al. 2014) to determine the mineralisation of BAM by free and immobilised MSH1 cells. Mineralisation experiments used MSN medium and were initiated by adding 250 µL of a stock solution containing unlabelled and ^14^C-labelled BAM (10,000 DPM) to reach a final concentration of 10 µg L^−1^ or 5 mg L^−1^ BAM in each flask. OD_600 nm_ measurements were used to determine the bacterial growth phase. Cells were harvested by centrifugation at 10,850×*g* for 10 min, and washed three times in MSN. MSH1 cells were re-suspended in MSN and 10^5^ cells were added to each flask. Finally, a small vial containing 2 mL 1 M NaOH was placed in the flasks to capture the ^14^CO_2_ produced. At regular intervals, the NaOH was replaced by fresh solution and mixed with 10 mL of OptiPhase ‘HiSafe’ 3 (Wallac, Scintillation Products, Skovlunde, Denmark) liquid and counted for 10 min on a liquid scintillation counter (Tri-Carb 2810, PerkinElmer, Skovlunde, Denmark).

## Results

### Characterisation of MSH1 and PM2 beads

#### Basic bead characteristics

The formulation of MSH1 gave reproducible, uniform and round-shaped beads approximately 1.25 mm in diameter as determined by microscopy. The beads had an average weight of 0.08 g (standard deviation of ±0.005 g) and a dry matter content of ~95 %. The viable cell number of MSH1 beads was 3–6 × 10^9^ CFU g^−1^ with an average of 4.4 × 10^9^ CFU g^−1^. The cell content of MSH1 powder was 2–5 × 10^9^ CFU g^−1^ with an average of 3.4 × 10^9^ CFU g^−1^. The average amount of viable PM2 cells varied between 0.32 and 2.3 × 10^9^ CFU g^−1^ bead with an average of 1.4 × 10^9^ CFU g^−1^ bead compared to MSH1 bead and powder.

#### Viability of bead cells over time

**Fig. 1:**
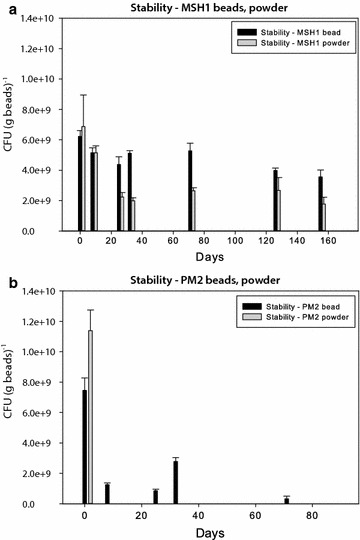
Viability of MSH1 (**a**) and PM2 (**b**) in powder and beads over time. Stability was measured in colony-forming unit per gram powder or beads at regular intervals over a period of about 5 months. Each data point is a mean of triplicates, with standard deviations shown with *errors bars*

The viability of MSH1 beads over time was investigated over a period of 5 months (Fig. 1a). Experiments showed that MSH1 beads retained ~60 % of the initial contents of viable cells. MSH1 powder, however, lost significantly more viable cells over 5 months, retaining only ~30 % of the initial CFU g^−1^ bead. The bioaugmentation experiments were therefore exclusively carried out using MSH1 beads. The storage stability of PM2 beads and powder was also investigated for comparison. PM2 powder did not contain viable cells and PM2 beads lost ~95 % of the initial CFU/g bead after ~72 days (Fig. 1b). The bioaugmentation experiments were therefore only conducted with MSH1 beads.

#### Correlation between viable cells and optical density

An OD/CFU correlation was determined for MSH1 and used to ensure that the bioremediation experiments were initiated with similar amounts of free and immobilised MSH1 cells. Immobilised cells were routinely tested after production for viability.

### Bioaugmentation tests with MSH1 beads

**Fig. 2:**
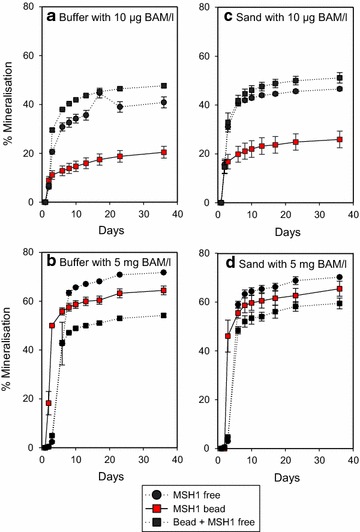
Mineralisation of ^14^C-*ring*-labelled BAM to ^14^CO_2_. BAM mineralisation with free and formulated MSH1 in buffer (**a**, **b**) and sand (**c**, **d**) expressed as ^14^C-BAM mineralised to ^14^CO_2_. Each data point is a mean of triplicates, with standard deviations shown with *errors bars*

Mineralisation of BAM with free MSH1 cells and MSH-containing beads was measured in buffer and sand with two different BAM concentrations (Fig. 2). There was stimulation in the initial mineralisation rate with MSH1 beads where there was a high BAM concentration (5 mg L^−1^) in both the buffer and sand (Fig. 2b, d). This positive effect, however, was not observed with the MSH1 beads with the low BAM concentration (10 µg L^−1^) (Fig. 2a, c). Free cells mineralised more BAM (about 40–50 % ^14^CO_2_ in  % of added ^14^C-BAM) than MSH1 beads at low BAM concentrations (around 20–25 % ^14^CO_2_) (Fig. 2a, c). With the high BAM concentration, however, the MSH1 beads and free cells produced a comparable extent of ^14^CO_2_ from ^14^C-BAM mineralisation, with 55–70 % ^14^C-BAM mineralised to ^14^CO_2_ during the experiment (Fig. 2b, d).

**Fig. 3:**
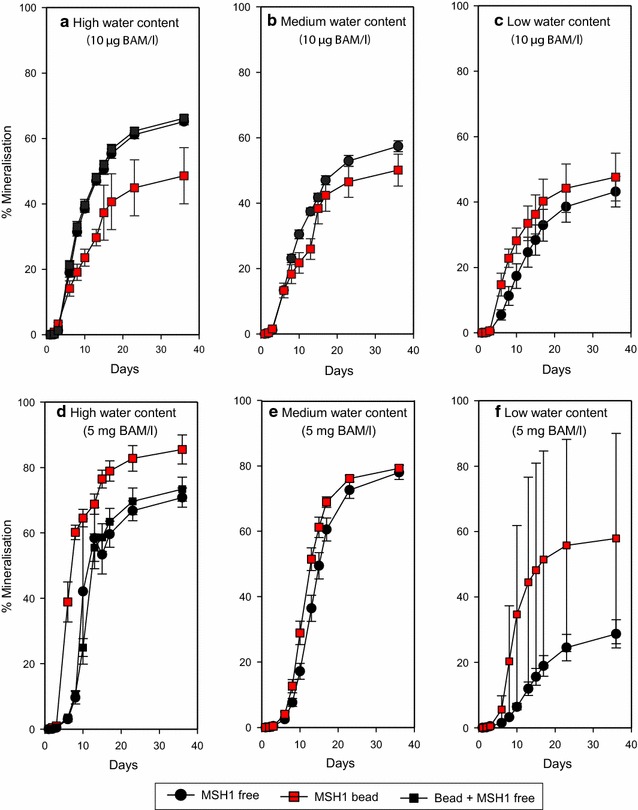
Mineralisation of ^14^C-*ring*-labelled BAM to ^14^CO_2_. BAM mineralisation with free and formulated MSH1 in soil with high (**a**, **d**), medium (**b**, **e**) and low (**c**, **f**) water contents. Each data point is a mean of triplicates, with standard deviations shown with *errors bars*

BAM mineralisation was also studied in soil with different water contents in order to compare the beads and the free MSH1 cells (Fig. 3). With the high BAM concentration (5 mg L^−1^), the MSH1 beads induced a slightly higher final level of mineralisation and a faster initial mineralisation rate compared to the free cells (Fig. 3d, e, f). The final mineralisation level of the low BAM concentration with MSH1 beads in soils with different moisture contents was approximately 50 % (Fig. 3a, b, c) and as high as 80 % with the high BAM concentration (Fig. 3d, e, f). The low water contents reduced BAM mineralisation with both free and formulated cells, and a tendency was apparent towards the beads performing better than the free cells (Fig. 3f).

## Discussion

MSH1 beads were capable of initiating BAM mineralisation in all the tested bioaugmentation scenarios. Earlier work by Sørensen et al. (2007) shows that MSH1-free cells perform better at mineralising high concentrations of BAM than at a low concentration of BAM, when evaluated based on the percentage of added ^14^C-BAM mineralised to ^14^CO_2_. This is in line with the present study’s findings, showing a general stimulation of the mineralisation extent when MSH1 beads are used at a high concentration of BAM (5 mg L^−1^) compared to the lower BAM concentration. The remaining part of the ^14^C-labelled BAM not mineralised to ^14^CO_2_ is likely incorporated into biomass as described previously by Sørensen et al. (2007). It was interesting, however, that the initial mineralisation rate of BAM was similar or, more frequently, even higher when MSH1 beads were challenged with high BAM concentrations in sand, buffer or soil. The initial mineralisation rate could be a crucial parameter in field remediation, as the flow rate of the passing water would determine the contact time between the degraders and BAM. Based on these evaluation criteria, further optimisation of the beads would appear relevant, especially when dealing with high BAM concentrations.

MSH1 beads showed no advantage over MSH1-free cells, however, when applied in sand and buffer at low concentrations of BAM using the mineralisation extent as an evaluation point. Here the initial mineralisation rate appeared similar, but the final extent of mineralisation was much lower for the MSH1 beads. In soil, the beads performed in approximately the same way as the free cells. It can be speculated that stress due to low water concentrations in soil with low water contents could be one of the reasons for the slightly better performance of MSH1 beads at low BAM concentrations. In the laboratory, it was also observed that beads dissolved more slowly under dry conditions in soil, which means that they could act as slow release carriers pulsing viable cells into the surrounding soil over a longer period of time.

Trehalose as a protecting agent for cell walls during freeze drying has been widely described in literature for yeast and bacterial strains (Leslie et al. 1995; Linders et al. 1997; Conrad et al. 2000; Carvalho et al. 2004). Trehalose was used successfully in this study for the formulation of the degrader strain *Aminobacter* sp. MSH1, but was less efficient in combination with the formulation method applied to the degrader strain *Sphingomonas* sp. PM2. PM2 beads lost almost 85 % of the viable cell numbers measured in CFU/g bead after 72 days. Nevertheless, the durability of the freeze-dried PM2 was enhanced significantly compared to the powdered formulation, which lost the activity measured in CFU g^−1^ bead completely after 2 days. This phenomenon was not investigated further in the current study, and consequently PM2 was not used for the bioremediation experiment. It is known that the protecting effect of trehalose can be more pronounced in some strains than in others (Carvalho et al. 2004). *Escherichia coli* and *Bacillus thuringiensis*, for example, are well protected during freeze drying and storage, whereas no effect of trehalose has been exhibited on *Lactobacillusplantarum* activity following drying (Leslie et al. 1995; Linders et al. 1997; Carvalho et al. 2004).

It has previously be found, that the most stable state for microorganisms is either frozen or in a dry matrix such as freeze-dried powder (Conrad et al. 2000). It is known that trehalose may act as a substrate for many microorganisms (Higashiyama 2002). However, no growth of MSH1 and PM2 was observed when trehalose was supplied as the sole carbon and energy source (results not shown). Besides being a potential substrate, trehalose can also protect microorganisms against various stresses including dryness and freezing (Higashiyama 2002). Trehalose has been shown to stabilise membranes and other groups of bio-molecular under extreme environmental conditions (Higashiyama 2002). Trehalose has a wide thermo- and pH-stability making it an appropriate cell protectant under many environmental conditions (Higashiyama 2002; Sapir and Harries 2011).

The formulated MSH1 beads are light, easy to transport and have a stable and large amount of viable cells for at least 6 months. This opens up the possibility of producing competent MSH1 beads away from the place of application at a convenient point in time. Beads could then easily be transported and applied for bioremediation purposes at different sites. The advantage of formulated MSH1 beads over more classical carrier strategies is that no waste carrier material remains to exacerbate environmental problems. Furthermore, the production of suitable carrier materials and particles is time consuming and an additional financial expense. Experience shows that up-scaling the production of carrier particles can be difficult, which makes the technology less convincing. Trehalose, however, is easily degradable and, to the authors’ knowledge, is not harmful to soil or water systems. Additionally, nutrients and other additives can easily be included to the formulation protocol developed here if additives were needed for the sustained activity of certain degrader strains in polluted environments.
